# Biological characterization and genomic analysis of a novel methicillin-resistant *Staphylococcus aureus* phage, SauPS-28

**DOI:** 10.1128/spectrum.00295-23

**Published:** 2024-01-09

**Authors:** Peisong Zhao, Wenli Zhao, Xin Zhai, Yulin He, Wei Shu, Guanhua Qiao

**Affiliations:** 1 Department of Microbiology, School of Basic Medicine, Guilin Medical University, Guilin, Guangxi, China; 2 Key Laboratory of Pathogenic Biology, Guilin Medical University, Guilin, Guangxi, China; 3 Department of Medical Laboratory, Handan Central Hospital, Handan, Hebei, China; 4 Department of Pharmacology, School of Basic Medical Sciences, Capital Medical University, Beijing, China; 5 Office of Health Insurance, The Fifth Affiliated Hospital of Zhengzhou University, Zhengzhou, China; 6 College of Intelligent Medicine and Biotechnology, Guilin Medical University, Guilin, Guangxi, China; 7 Key Laboratory of Environmental Exposomics and Entire Lifecycle Heath, Guilin Medical University, Guilin, Guangxi, China; Yangzhou University, Yangzhou, Jiangsu, China

**Keywords:** phage, methicillin-resistant *Staphylococcus aureus*, biological properties, genomic analysis, proteomic analysis, phage therapy

## Abstract

*Staphylococcus aureus,* a representative gram-positive bacterium, is a common infectious pathogen widely present in the natural environment. The increasing application of antibiotics is witnessing an increment in the number of clinically resistant strains (such as methicillin-resistant *S. aureus* [MRSA]), which has posed a great challenge to antimicrobial therapy. In this study, a novel MRSA phage, SauPS-28, was isolated from the lake water of the Guangxi Zhuang Autonomous Region. This phage has an incubation period of approximately 30 min, a lysis period of approximately 40 min, and a burst size of approximately 25 PFU/cell. The isolated phage exhibited good biological stability at a pH range of 6.0–9.0 and temperature range of 4°C–37°C. In addition, the identification of an elongated tail using transmission electron microscopy confirmed that SauPS-28 belongs to the long-tailed phage family. Whole-genome sequencing analysis revealed that SauPS-28 has a 43,286-bp-long genome with 31.03% G + C content. Moreover, SauPS-28 exhibited 95.69% sequence identity with ECel-2020k, while the query coverage was only 66%, which is a newly discovered phage. Whole-genome functional annotation results revealed that SauPS-28 had 68 open reading frames (ORFs). Of these, 30 ORFs are unknown proteins. The results suggest that SauPS-28 could be a lysogenic phage strain. This study thus provides preliminary data to conduct further in-depth analysis of the mechanism of phage–host interaction and provides a reference value for phage therapy.

**IMPORTANCE:**

In recent years, drug-resistant bacterial infections have become increasingly serious. As a kind of virus with the ability to infect and lyse drug-resistant bacteria, phage is expected to be a new therapeutic method. In this study, we isolated and purified a new methicillin-resistant *Staphylococcus aureus* bacteriophage SauPS-28, studied a series of biological characteristics of the bacteriophage, analyzed the genome and structural proteome data of the bacteriophage, and provided reference data for further study of the interaction mechanism between bacteriophage and host bacteria and promoted new antibacterial strategies.

## INTRODUCTION

*Staphylococcus aureus* is a common gram-positive pathogen that carries various virulence factors and can cause various diseases (1). It can cause infectious diseases, such as skin infections, arthritis, sepsis, or septicemia (2–6), and toxigenic diseases, such as food poisoning, scald-like skin syndrome, and toxic shock syndrome (7–11). With the discovery and indiscriminate use of antibiotics, various drug-resistant bacteria have gradually emerged. The emergence of drug-resistant bacterial strains such as methicillin-resistant *S. aureus* (MRSA) has posed a great challenge to clinically anti-infective treatment. The search for novel therapeutics has become an urgent problem.

Phages are a class of viruses with prokaryotic bacterial hosts and are characterized by high diversity, abundance, and specificity (12–14). They are categorized as lysogenic and lytic. In 1921, phages were first discovered to possess the ability to lyse bacteria and were therefore used to treat bacterial infections (15). The discovery and extensive use of antibiotics thereafter contributed to the decline in phage therapy research. However, the recent increase in cases of antibiotic resistance in bacteria and the completely different bactericidal mechanisms from that of antibiotics have renewed interest in phage therapy (16–18). Lysogenic phages are diverse and widely distributed, with nearly half of the sequenced bacteria being lysogenic (19); however, their ability to kill bacteria is limited, and superinfectious immunity caused by gene integration also reduces the susceptibility of host bacteria to phages (20). Horizontal gene transfer (HGT) causes toxin genes, virulence island gene clusters, and antibiotic-resistant genes in bacteria, which make them more virulent and difficult to treat (21–27), so the first choice for phage therapeutic recommendations are strictly lytic phages that can kill the host quickly and effectively. However, this guideline was developed before efficient strategies of phage genome engineering. As high-throughput sequencing and synthetic biology advanced, the special advantages of lysogenic phages were gradually revealed. The rapid increase in the number of bacterial genome sequences in bioinformatics databases, the development of bioinformatics tools for genomic analysis and lysogenic phage marker gene (integrases, etc.) identification, and the abundance of lysogenic phages in bacterial genomes have made the detection and isolation of lysogenic phages easier, in particular for anaerobic and *in vitro* difficult-to-incubate pathogenic bacteria (28). Lytic mutant phage variants can be obtained by the mutagenesis of lysogenic phages and gene editing techniques, which could remove gene modules known to be involved in the establishment and maintenance of the lysogenic life cycle and genes for bacterial virulence in lysogenic phages, and then transform lysogenic phages into strictly lytic phages (29, 30). Lysogenic phages can have their host range and bactericidal potential altered by genetic engineering and recombination procedures (29). The use of designed lysogenic phages to transport synthetic gene networks that interfere with crucial bacterial intracellular processes has been made possible by advances in synthetic biology (31). These synthetic gene networks can cause cell death of the bacteria or convert them to be antibiotic sensitive (32). The focus on lysogenic phage utilization will help contribute to the development of more comprehensive bacterial treatments.

Several successful cases of phage application in models or clinical treatments have been reported, which fully validate the feasibility of phage therapy (33–37). For instance, Doub et al. and Schoeffel et al. reported the effectiveness of *S. aureus* phages in the treatment of recalcitrant MRSA-induced arthritis (34, 38). Teng et al. evaluated the effectiveness of phage treatment for mastitis in a mouse model of MRSA and demonstrated that MRSA colonization in the mammary gland was significantly inhibited (39). Chung et al. reported that lysogenic phage D3112 significantly reduced mortality in fly models infected with *Pseudomonas aeruginosa* either orally or via injection (40). Breyne et al. adopted a phage cocktail method to treat bovine mastitis and found a significantly reduced infection rate of bovine mastitis in mice, with improved pathologic changes and reduced bacterial counts (41). Meanwhile, phage antibiotic combination (PAC) therapy has become a research hotspot (42). Rodriguez et al. successfully cured MRSA-induced refractory chronic sinusitis (43). Both phage therapy and PAC therapy have shown the potential value of phages against drug-resistant bacterial infections, such as MRSA infections.

In this study, a novel strain of MRSA phage was isolated and identified from Guangxi Zhuang Autonomous Region. Preliminary studies on the biological properties of this novel strain, such as lysis ability, growth curve, and stability, were conducted, and preliminary analyses of the whole genome and proteome were conducted.

## RESULTS

### Morphological observation of phage SauPS-28

**Fig 1:**
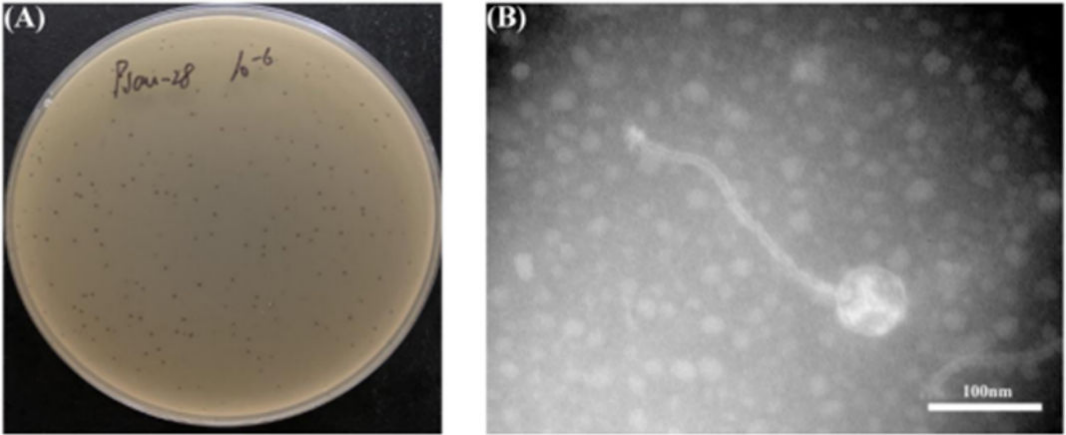
Bacterial plaque and TEM observation. (**A**) Double-layer agar assay of phage SauPS-28 with the host bacteria. (**B**) TEM observation of phage SauPS-28 with a positive polyhedral head structure, about 40 nm in diameter; showing an elongated tail of approximately 190 nm length and an overall length of approximately 230 nm. Scale bar = 100 nm.

A novel MRSA phage, SauPS-28, was isolated from the lake water of Guangxi Zhuang Autonomous Region, China, by using MRSA obtained from the Second Affiliated Hospital of Guilin Medical College as the host strain. Clear and round phage spots were observed on the double-agar plate (DAP), confirming the lytic ability of SauPS-28 (Fig. 1A). The morphological features of this phage were observed under a Hitachi TEM HT7700 at 80 kV. SauPS-28 was tadpole-shaped with a positive polyhedral head structure, approximately 40 nm in diameter. It had an approximately 190-nm-long slender tail and an overall length of approximately 230 nm, which was typical of T-series phages (Fig. 1B). According to the classification guidelines proposed by the International Committee on Taxonomy of Viruses, the morphology of phage SauPS-28 was consistent with the morphological characteristics of Siphoviridae: it is thus a new member of the Siphoviridae family (44).

### Host-range determination

SauPS-28, 22 MRSA strains, and the host range of SauPS-28 were detected using the DAP method (Table 1). Excluding the bacterial host, six MRSA strains were lysed by SauPS-28, and phage spots visible to the naked eye were formed on agar plates, which indicated that the phage had a wide host range.

**TABLE 1:** The host-range spectrum of the phage SauPS-28^*a*^

MRSA (No.)^*b*^	Resistance	Lysis ability	Provider	Isolation time
1	Penicillin, benzocillin, gentamicin, telithromycin, tetracycline, erythromycin, clindamycin, norfloxacin, moxifloxacin, chloramphenicol	N	Hospital	2019/04/26
2	Cefoxitin, ampicillin, penicillin, benzocillin, amoxicillin, clindamycin, erythromycin, tetracycline	Y	Hospital	2019/04/30
3	Cefoxitin, ampicillin, penicillin, benzocillin, amoxicillin, erythromycin, clindamycin, ciprofloxacin	N	Hospital	2019/05/03
4	Cefoxitin, ampicillin, penicillin, benzocillin, amoxicillin, erythromycin, clindamycin	Y	Hospital	2019/05/11
5	Cefoxitin, ampicillin, penicillin, benzocillin, amoxicillin, erythromycin, clindamycin	N	Hospital	2019/05/17
6	Gentamicin, tobramycin, cefoxitin, ampicillin, penicillin, benzocillin, amoxicillin, clarithromycin, erythromycin, sulfadoxine, methicillin	N	Hospital	2019/05/18
7	Cefoxitin, ampicillin, penicillin, benzocillin, amoxicillin, clarithromycin, erythromycin	N	Hospital	2019/05/27
8	Cefoxitin, ampicillin, penicillin, benzathine, amoxicillin, methicillin, teicoplanin, erythromycin, tetracycline	N	Hospital	2019/06/03
9	Cefoxitin, ampicillin, penicillin, benzocillin, amoxicillin, sulfadoxine, methicillin, clindamycin, erythromycin, rifampicin	N	Hospital	2019/06/07
10	Cefoxitin, ampicillin, penicillin, benzocillin, amoxicillin, compound sulphonamide, methicillin, rifampicin	N	Hospital	2019/06/09
11	Cefoxitin, ampicillin, penicillin, benzocillin, amoxicillin, clindamycin, erythromycin	Y	Hospital	2019/06/08
12	Cefoxitin, ampicillin, penicillin, benzocillin, amoxicillin, sulfadoxine, methicillin, rifampicin, clindamycin, erythromycin	N	Hospital	2019/06/17
13	Cefoxitin, ampicillin, penicillin, benzocillin, amoxicillin, clindamycin, erythromycin, methicillin, rifampicin	N	Hospital	2019/06/24
14	Amikacin, tobramycin, cefoxitin, ampicillin, penicillin, benzocillin, amoxicillin, erythromycin, tetracycline	N	Hospital	2019/07/01
15	Cefoxitin, ampicillin, penicillin, benzocillin, amoxicillin, erythromycin, clindamycin	N	Hospital	2019/07/13
16	Amikacin, cefoxitin, ampicillin, penicillin, benzocillin, amoxicillin, clindamycin, erythromycin	Y	Hospital	2019/07/25
17	Gentamicin, cefoxitin, ampicillin, penicillin, benzocillin, amoxicillin, teicoplanin, vancomycin, clindamycin, quinupristin, mupirocin, rifampicin, tetracycline	N	Hospital	2019/07/25
18	Cefoxitin, ampicillin, penicillin, benzocillin, amoxicillin, clindamycin, erythromycin	Y	Hospital	2019/08/11
19	Cefoxitin, ampicillin, penicillin, benzocillin, amoxicillin, clindamycin, erythromycin	Y	Hospital	2019/08/13
20	Cefoxitin, ampicillin, penicillin, benzocillin, amoxicillin, methicillin, clindamycin, erythromycin	Y	Hospital	2019/08/15
21	Gentamicin, tobramycin, cefoxitin, ampicillin, penicillin, benzocillin, amoxicillin, erythromycin, tetracycline, methicillin, clindamycin	N	Hospital	2019/08/15
22	Gentamicin, tobramycin, cefoxitin, ampicillin, penicillin, bumazacillin, amoxicillin, fosfamide, methomyl, clindamycin, erythromycin, quinupristin, ciprofloxacin, rifampicin, tetracycline	N	Hospital	2019/8/20

^*a*^ Negative results are indicated as "N" and positive results as "Y".

^*b*^ MRSA, methicillin-resistant *Streptococcus aureus*.

### Infection plural and growth curve

Most phage spots were observed on the DAP when SauPS-28 was mixed with the bacterial host at the optimal multiplicity of infection (MOI) ratio (Table 2). SauPS-28 produced the highest titer of daughter phages when it was mixed cultured with the host strain at the MOI ratio of 0.01. Three sets of parallel experiments were conducted for different MOI ratios, and the average of different results was used to plot the table.

**TABLE 2:** Phage SauPS-28 optimal multiplicity of infection (MOI)

Number	Phage titer (PFU/mL)	Bacterial concentration (CFU/mL)	MOI	Titer (PFU/mL)
1	10^9^	10^8^	10	3.66 × 10^7^
2	10^8^	10^8^	1	8.40 × 10^7^
3	10^7^	10^8^	0.1	1.03 × 10^10^
4	10^6^	10^8^	0.01	2.01 × 10^10^
5	10^5^	10^8^	0.001	8.17 × 10^9^

**Fig 2:**
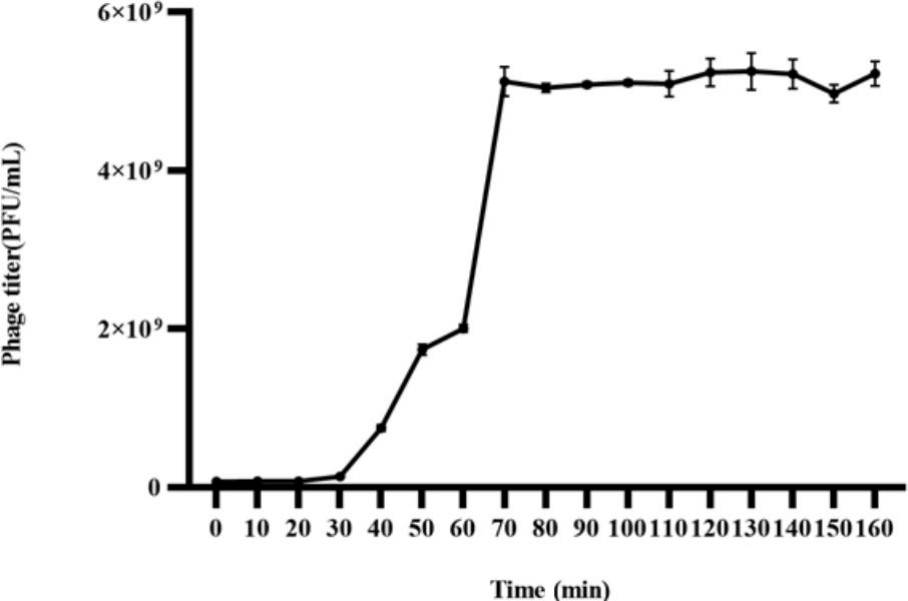
The growth curve of phage SauPS-28. The latency period of the phage is approximately 30 min, the lysis period is approximately 40 min, and the burst amount is approximately 25 PFU/cell. The experiment was repeated thrice, and the error line shows the mean ± SD.

SauPS-28 and the host strain were mixed at the MOI ratio of 0.01 and incubated at 37°C and 180 rpm, and the growth curve of SauPS-28 was plotted every 10 min by using the DAP method to detect the phage titer (Fig. 2). SauPS-28 entered the lytic phase after a latent period of approximately 30 min. The phage titer increased rapidly within 30–70 min, followed by a plateau phase, which indicated a lytic phase of approximately 40 min. Phage burst is the ratio of the total daughter phage titer released from infected cells at the beginning of the plateau phase to the initial number of the infected host during the latent phase, with a burst of approximately 25 PFU/cell. The experiments were repeated thrice, and the error lines were presented as the mean ± SD.

### Stability study of phage SauPS-28

**Fig 3:**
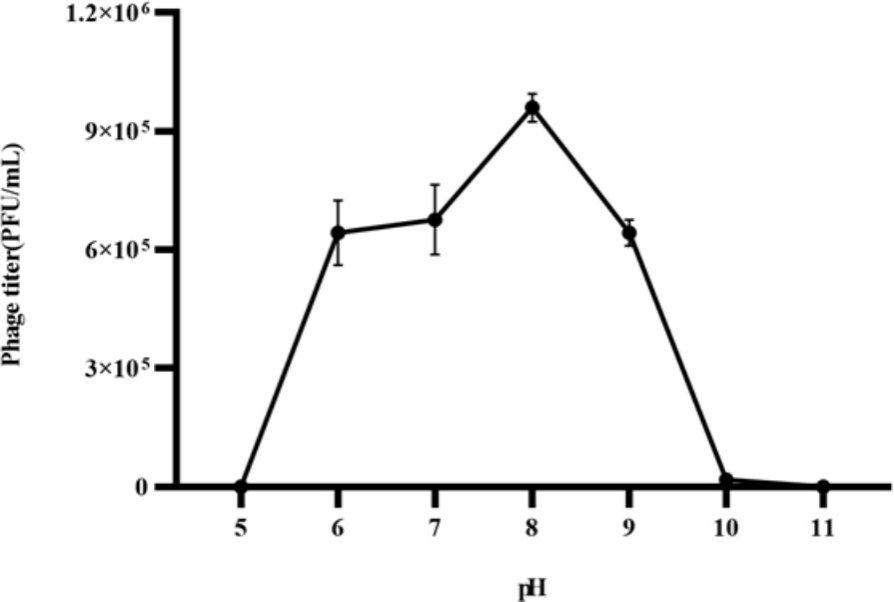
Acid–base stability of the phage SauPS-28. The optimum pH of phage SauPS-28 is 8, and the activity completely disappears when the pH is <5 or >10. The experiment was repeated thrice, and the error line shows the mean ± SD.

**Fig 4:**
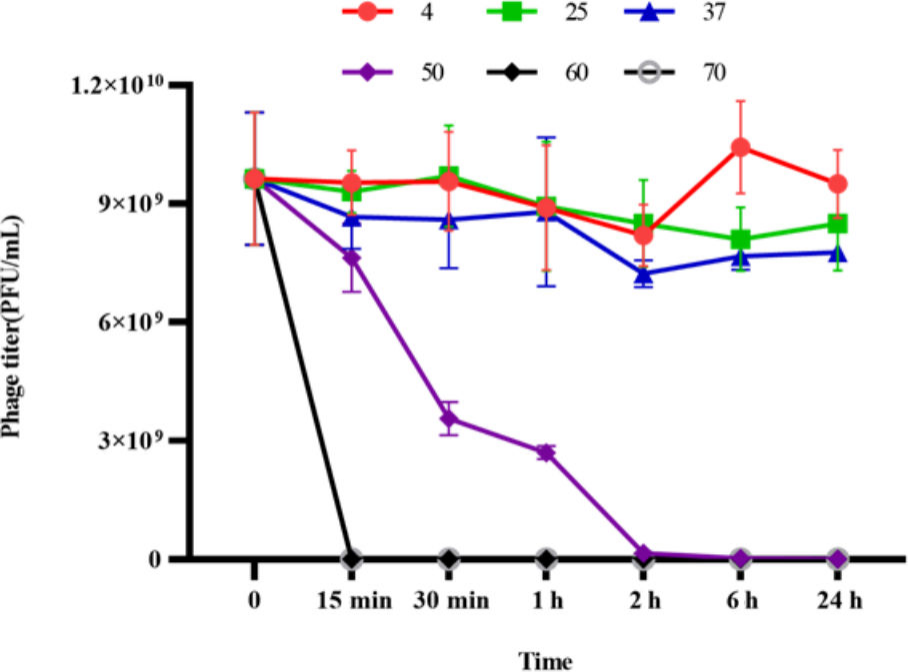
Thermal stability of phage SauPS-28. Phage SauPS-28 was incubated at 4°C, 37°C for 24 h and the activity was basically stable; at 50°C for 15 min, the titer of phage SauPS-28 decreased continuously and it became completely inactivated at 6 h; at 60°C or 70°C, phage SauPS-28 was completely inactive. The experiment was repeated thrice, and the error line shows the mean ± SD.

SauPS-28 was incubated in different buffer pairs (pH = 5, 6, 7, 8, 9, 10, and 11) at 37°C for 1 h. The DAP method was used to assay the titers of phage suspensions at different pH values (Fig. 3). The optimum pH of SauPS-28 was 8, and the phage activity was completely lost at a pH below 5 or above 10. The experiment was repeated thrice, and the error line was presented as the mean ± SD. SauPS-28 thermal stability experiments were performed at different temperatures (4°C, 25°C, 37°C, 50°C, 60°C, and 70°C) and continuous incubation for 24 h. The DAP was used to assay SauPS-28 at different temperatures and different time points (0 min, 15 min, 30 min, 1 h, 2 h, 6 h, and 24 h), respectively, of phage titers (Fig. 4). The results revealed that SauPS-28 activity was stable at 4°C and 24 h at 37°C. At 50°C for 15 min, the titer of SauPS-28 decreased continuously, and SauPS-28 was completely inactivated at 6 h. At 60°C or 70°C, SauPS-28 was completely inactive. The experiment was repeated thrice, and the error line was presented as the mean ± SD.

### Restriction digest profile of phage SauPS-28

**Fig 5:**
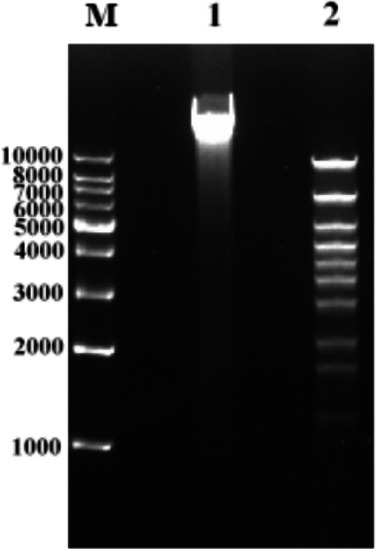
The restriction digest profile of phage SauPS-28. Restriction endonuclease EcoR I was digested for 4 h, and multiple bands were separated using 0.7% agarose gel electrophoresis, indicating that SauPS-28 is a linear double-stranded DNA.

The total nucleic acid of SauPS-28 was extracted using the Phage Genomic DNA Extraction Kit. Enzymatic identification was performed with restriction endonuclease *EcoR I* (Fig. 5). Lane M represents the marker, Lane 1 represents SauPS-28 total nucleic acid, and Lane 2 represents the sample after restriction endonuclease digestion. The genome was digested by *EcoR I* with multiple bands, indicating that SauPS-28 is linear double-stranded DNA.

### Phage SauPS-28 proteome analysis

**Fig 6:**
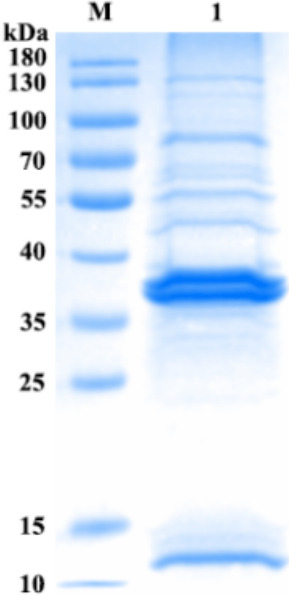
Separation of phage SauPS-28 structural proteins using SDS-PAGE gel. Phage SauPS-28 was treated with PEG8000, quantified using BCA, and denatured at 100°C, followed by separation on SDS-PAGE and staining with Coomassie Brilliant Blue-R250. The molecular weight of the ORFs corresponding to their functional annotation results was, in descending order, phage tail tape measure protein, terminase, phage major capsid protein, phage portal protein, and the hypothetical protein. The molecular masses of the proteins are marked on the far left panel.

SauPS-28 was concentrated with PEG8000 and then, according to the BCA standard protein profile, ultrasonically broken and denatured at 100°C. SDS-PAGE was performed using Coomassie Brilliant Blue R-250 staining. The results revealed at least six more obvious protein bands in the SDS-PAGE gel (Fig. 6), corresponding to the results of functional annotation of open reading frames (ORFs) (Table 3). The predicted functions of the proteins encoded using the ORFs in the order of molecular weights from the largest to the smallest were 75.4 kDa (ORF29, encoding phage tail tape measure protein), 55.1 kDa (ORF16, encoding terminase), 43.7 kDa (ORF20, encoding phage major capsid protein), 38.8 kDa (ORF18, encoding phage portal protein), 36.4 kDa (ORF31, encoding hypothetical protein), and 11.4 kDa (ORF22, encoding phage head–tail connector protein). Among them, ORF29, ORF20, ORF18, and ORF22 encoded structural proteins of phage SauPS-28, and ORF16 is a terminal enzyme. ORF31 is a hypothetical protein, and its potential function needs to be determined.

**TABLE 3:** Open reading frame (ORF) analysis of the phage SauPS-28 genome

ORFs	Start	End	Dire.	MW (kDa)	Predicted protein function
ORF01	2	133	+	7.1	DUF3113 family protein
ORF02	134	490	+	13.4	hypothetical protein
ORF03	494	736	+	9.5	hypothetical protein
ORF04	742	999	+	9.2	phage protein
ORF05	992	1,501	+	8.1	hypothetical protein
ORF06	1,538	1,783	+	9.0	hypothetical protein
ORF07	1,780	1,980	+	7.3	hypothetical protein
ORF08	1,980	2,222	+	5.1	phage protein
ORF09	2,253	2,648	+	14.0	hypothetical protein
ORF10	2,645	2,818	+	6.9	transcriptional regulator
ORF11	2,818	3,018	+	7.1	DUF1514 family protein
ORF12	3,041	3,511	+	17.3	hypothetical protein
ORF13	3,608	4,078	+	9.2	nucleoside triphosphate pyrophosphohydrolase protein
ORF14	4,094	4,438	+	13.6	HNH endonuclease
ORF15	4,566	5,036	+	10.4	phage terminase small subunit
ORF16	5,036	6,730	+	55.1	phage terminase
ORF17	6,711	6,947	+	10.0	hypothetical protein
ORF18	6,950	8,224	+	38.8	phage portal protein
ORF19	8,199	8,783	+	18.7	phage prohead protease
ORF20	8,871	10,106	+	43.7	phage major capsid protein
ORF21	10,143	10,301	+	5.8	hypothetical protein
ORF22	10,310	10,642	+	11.4	phage head–tail connector protein
ORF23	10,632	10,964	+	12.2	head–tail adaptor protein
ORF24	10,964	11,341	+	12.8	phage protein
ORF25	11,338	11,727	+	14.3	hypothetical protein
ORF26	11,720	12,673	+	21.1	Ig-like domain-containing protein
ORF27	12,739	13,185	+	16.4	hypothetical protein
ORF28	13,260	13,367	+	5.9	Uncharacterized protein
ORF29	13,423	18,090	+	75.4	phage tail tape measure protein
ORF30	18,090	19,580	+	19.1	phage tail protein
ORF31	19,596	23,276	+	36.4	hypothetical protein
ORF32	23,263	23,415	+	5.6	hypothetical protein
ORF33	23,462	23,749	+	10.6	hypothetical protein
ORF34	23,807	24,103	+	11.7	DUF2951 domain-containing protein
ORF35	24,641	24,943	+	8.0	Holin
ORF36	24,955	26,409	+	20.1	*N*-acetylmuramoyl-l-alanine amidase
ORF37	26,654	26,806	+	5.6	hypothetical protein
ORF38	26,974	27,174	+	7.3	hypothetical protein
ORF39	27,521	28,570	+	40	hypothetical protein
ORF40	28,578	28,850	+	10.0	predicted protein
ORF41	28,847	29,530	+	25.2	hypothetical protein
ORF42	29,499	29,645	+	5.1	hypothetical protein
ORF43	31,039	29,834	−	21.9	site-specific integrase
ORF44	31,988	31,233	−	5.6	toxin-antitoxin system, toxin component, MazF family
ORF45	32,206	32,024	−	6.7	hypothetical protein
ORF46	33,108	322,51	−	5.5	restriction endonuclease
ORF47	33,836	33,120	−	26.9	repressor-like protein
ORF48	33,928	34,242	+	8.0	hypothetical protein
ORF49	34,258	35,055	+	29.4	phage containing protein
ORF50	35,069	35,485	+	15.3	transcriptional regulator
ORF51	35,562	35,801	+	8.8	hypothetical protein
ORF52	36,128	36,454	+	12.0	hypothetical protein
ORF53	36,487	36,669	+	8.0	hypothetical protein
ORF54	36,699	36,962	+	10.2	helix-turn-helix domain-containing protein
ORF55	37,104	36,994	−	4.0	hypothetical protein
ORF56	37,228	37,530	+	10.8	DUF2482 family protein
ORF57	37,535	37,795	+	9.9	DUF1108 family protein
ORF58	37,808	38,344	+	17.6	host-nuclease inhibitor Gam family protein
ORF59	38,345	38,995	+	17.4	ERF family protein
ORF60	389,92	39,435	+	16.2	single-stranded DNA-binding protein
ORF61	39,447	40,115	+	23.8	hypothetical protein
ORF62	40,717	40,301	−	15.3	hypothetical protein
ORF63	40,762	41,523	+	28.1	phage containing protein
ORF64	41,536	42,321	+	26.9	ATP-binding protein
ORF65	42,318	42,476	+	5.8	hypothetical protein
ORF66	42,489	42,710	+	8.5	DUF3269 family protein
ORF67	42,700	43,125	+	13.4	hypothetical protein
ORF68	43,130	43,285	+	7.2	DUF3113 family protein

### Genome annotation and function of the phage SauPS-28

**Fig 7:**
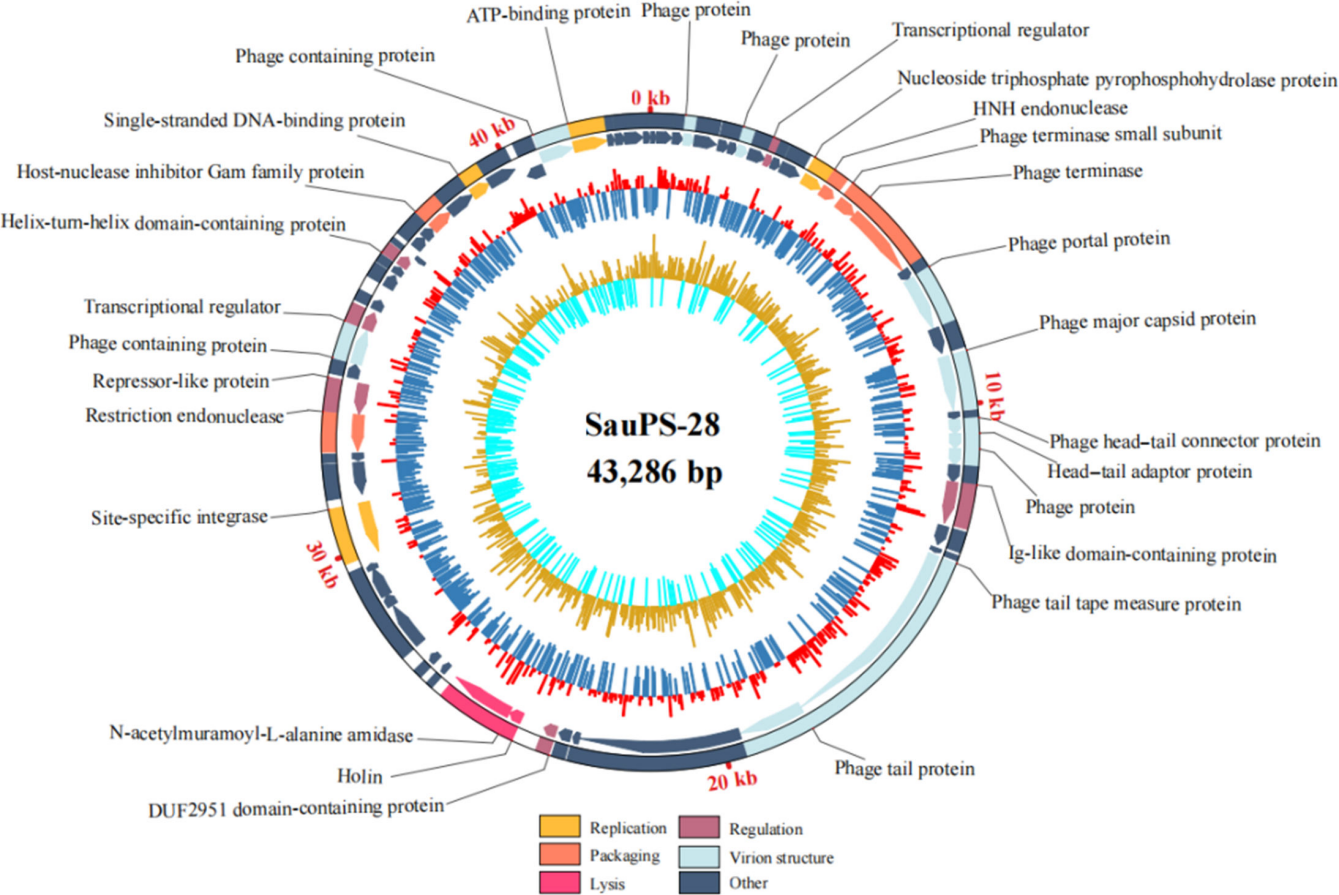
Circos plot of the SauPS-28 genome. The outermost ring displays the distribution of different types of genes on the genome, with clockwise arrows representing genes in the positive direction in the genome and the counterclockwise table genomes representing genes in the negative direction. The red bars indicate the regions of the genome with higher than average GC content, while the dark blue bars indicate the regions of the genome with lower than average GC content. The yellow bars indicate the regions of the genome with positive GC skew, while the sky-blue bars indicate the regions of the genome with negative GC skew.

Phage SauPS-28 has a linear double-stranded DNA with a 43,286-bp-long genome and a GC content of 31.03% (GenBank accession number: BankIt2665638 Seq1 OQ588745). The whole genome sequence of SauPS-28 was analyzed using BLASTn from the NCBI non-redundant DNA database. The whole genome sequence identity of SauPS-28 and phage ECel-2020k (CP062426.1) was 95.69%, while the query coverage was only 66%, thus indicating that the genome sequence of SauPS-28 was relatively new. Functional annotation using RAST revealed that SauPS-28 contained 68 ORFs. Of them, 53 ORFs had ATG as the start codon (77.94%), 9 ORFs had GTG as the start codon (13.2%), 5 ORFs had TTG as the start codon (7.3%), and 1 ORF had GTT as the start codon. A total of 61 ORFs were forward transcribed and 7 ORFs were reverse transcribed. The functional proteins encoded using the ORFs were analyzed using BLASTp and divided into six modules: virion structure, regulation, packaging, replication, lysis, and others (Table 3, Fig. 7). Some ORFs encoded proteins as part of the virion structure, including the phage protein, phage portal protein, phage major capsid protein, phage head–tail connector protein, and head–tail adaptor protein. Some ORFs encoded proteins involved in regulation (i.e., transcriptional regulator, Ig-like domain-containing protein, DUF2951 domain-containing protein, repressor-like protein, and helix-turn-helix domain-containing protein). Some ORFs encoded proteins involved in the packaging (i.e., HNH endonuclease, phage terminase small subunit, phage terminase, restriction endonuclease, and host-nuclease inhibitor Gam family protein). Some ORFs encoded proteins involved in replication, including nucleoside triphosphate pyrophosphohydrolase protein, site-specific integrase, single-stranded DNA-binding protein, and ATP-binding protein). Some ORFs encoded proteins involved in lysis (i.e., holin and *N*-acetylmuramoyl-l-alanine amidase), which are responsible for most of the lysis of bacterial strains. The module categorized as other included the hypothetical protein, DUF3113 family protein, DUF1514 family protein, DUF2482 family protein, DUF1108 family protein, ERF family protein, DUF3269 family protein, DUF3113 family protein, as well as the toxin–antitoxin system, toxin component, MazF family, and phage prohead protease. This indicates that SauPS-28 is a lysogenic phage. The potential functions of hypothetical proteins in SauPS-28 need to be further investigated. No tRNA has been found in the SauPS-28 genome.

### Phylogenetic tree analysis

**Fig 8:**
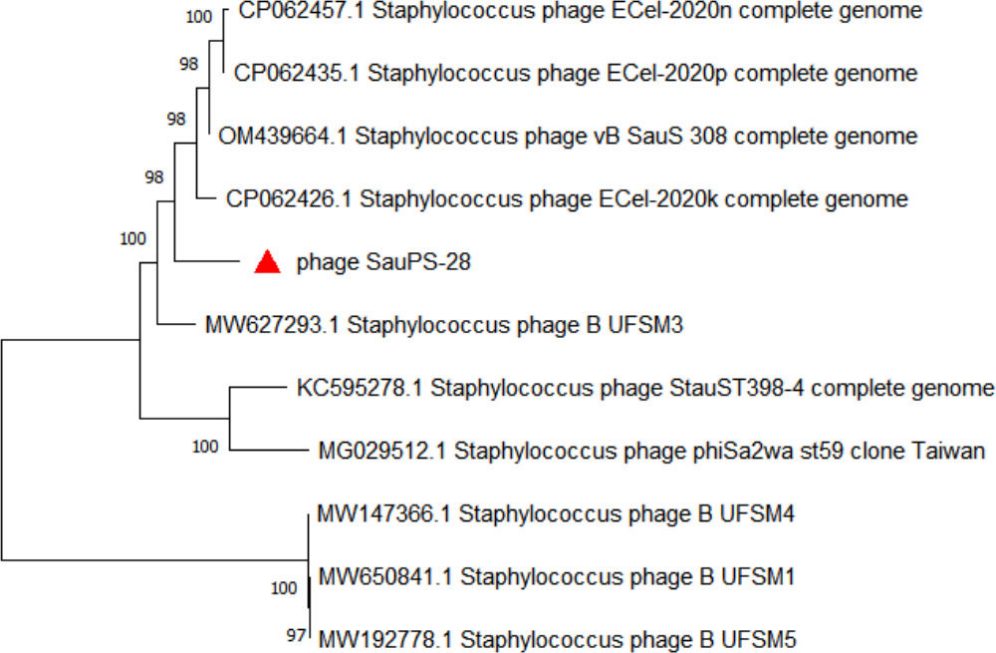
Phylogenetic tree of the whole genome sequences of SauPS-28 phage and its related phages. The genomic sequences of these phages were compared based on the ClustalW comparison algorithm, ML, and a phylogenetic tree with 1000 bootstrap repeats using MEGA-X software. SauPS-28 is marked in red.

To further analyze the evolutionary origin of SauPS-28, the whole genome sequence was analyzed using MEGA-X software to construct a phylogenetic tree (Fig. 8). According to the results, SauPS-28 belonged to the same group as phage ECel-2020n, phage ECel-2020p, phage vB SauS 308, and phage ECel-2020k, but in different branches. In addition, SauPS-28 was distant from all other phages. The results indicated that SauPS-28 was a new phage strain, and bootstrap values of >70 indicated reliable results.

## DISCUSSION

The emergence of large numbers of multidrug-resistant bacteria poses a huge challenge to the treatment of bacterial infections, and phage therapy can be applied as the last-resort treatment as a compassionate medication approach when antibiotic therapies have demonstrated limited efficacy and it is difficult to reverse the disease (45, 46). Lytic phages have a diverse host range that are optimal for phage therapy. In this study, we obtained the long-tailed phage SauPS-28 by isolation of lytic phages, and the sequencing results revealed a genome of length 43 kb. Homology comparison identified a 95.69% sequence identity with the whole genome of phage ECel-2020k, with 66% query coverage, which implies that it was a new phage virulence strain. Moreover, the gene annotation results indicated that it encoded at least 68 ORFs. The discovery of the gene encoding site-specific integrase suggests that it may belong to a lysogenic phage.

Lysogenic phages can act as HGT vectors, and they act as a source of deleterious genes (e.g., virulence and antibiotic resistance) in the host bacterium through lysogeny, unlike lytic phages (21, 47–49). Therefore, performing the necessary genetic modifications and removing the corresponding deleterious genes is a prerequisite for lysogenic phage to use in phage therapy (48, 50). Genetically modified lysogenic phages have been applied in the treatment of infections caused by *Salmonella* (51) and *Mycobacterium* (52).

In this study, experiments conducted on phage isolation, purification, multiplication, growth curves, and thermal and acid–base stability relied on plaque-forming units (PFU) counts (which are the typical characteristics of lysogenic phages) and revealed no obvious anomalies, possibly caused by the lysogenic phenomena. This observation is contrary to the results implied by the presence of site-specific integrases in the genome, with the following possibilities: (i) at the molecular level, the presence of a gene-encoding integrase is not a sufficient condition for a phage to be identified as a lysogenic phage, and the sequencing results can only identify the presence of an integrase-coding sequence, which neither reflects the actual activity of integrase nor predicts the inevitable occurrence of lysogeny; (ii) host bacteria and bacteriophages may lack all genetic elements or protein factors necessary for integration due to genetic mutations. Based on these factors, the categorization of phages as either lysogenic or lytic may be both crude and debatable, considering the changes in integrase activity potentially brought about through genetic mutations. Lysogeny or lysis (a phenomenon or state of affairs) is more likely to be host-dependent because of the coupling of a specific bacterium and a specific phage; however, the manifestation of host dependence may not be appropriate as a basis for phage categorization. Therefore, this hypothesis needs to be further investigated and validated through subsequent trials to advance the current development in phage therapy.

## MATERIALS AND METHODS

### Bacterial strains and growth conditions

The clinical strains used in this study were obtained from the Second Affiliated Hospital of Guilin Medical College and stored at −80°C in Luria–Bertani (LB) medium and solid medium (Qingdao Haibo Biotechnology Co.)

### Isolation and purification of phage

The phage was isolated from the lake water of Guilin Medical College (Lingui District, Guangxi, China) and named SauPS-28. Isolation: 100 mL of the lake water was centrifuged at 10,000 ×*g* for 10 min at 4°C, and the precipitate was discarded. The supernatant was filtered using a 0.22-µm membrane (Millibol, Massachusetts, USA) and de-bacterized, followed by mixing with 100 mL of the LB broth and inoculation with MRSA at the logarithmic growth stage (OD600 about 0.6). Then, 1 mL of the bacterial broth was incubated at 37°C under 180 rpm shaking for 8 h, followed by centrifugation at 10,000 ×*g* for 10 min at 4°C and discarding of the precipitate. The supernatant was filtered through a 0.22-µm membrane to obtain the phage stock solution. Phage purification was performed using the DAP method. Briefly, the phage SauPS-28 stock solution series was diluted to 10^−8^, mixed with 100 µL dilution with 100 µL of the MRSA bacterial solution at each dilution gradient, and incubated at 37°C for 10 min. Then, semi-solid agar (warmed to 56°C) was added, mixed well, and poured onto the surface of the LB plates. After incubation of the plates at 37°C for approximately 13 h, the phage spots were enumerated on the plate. A sterile gun tip was used to pick clearer and smoother individual phage spots, which were then immersed in 1 mL of Tris–SM buffer, eluted with gentle shaking, and serially diluted for secondary screening. After four screenings, the phage spots of uniform size and typical characteristics were obtained.

### Phage multiplication, concentration, and preservation

In accordance with the method of Gavric et al. (38), albeit with slight modifications, a single phage spot was inoculated with 100 mL of a logarithmic growth-phase MRSA solution, incubated at 37°C under 180 rpm shaking for 8 h, followed by centrifugation at 10,000 ×*g* at 4°C for 10 min, and the discarding of the precipitate. The supernatant was then filtered through a 0.22-µm filter membrane to obtain the phage culture. DNase I and RNase A were added to a final concentration of 1 µg/mL to the culture and left to stand at 37°C for 10 min. After which, sterile NaCl solution was used to make a final concentration of 1 mol/L, and the mixture was shaken well to mix on an ice bath for 1 h, followed by centrifugation at 8,000 ×*g* for 30 min at 4°C, addition of polyethylene glycol (PEG) 8000 to the supernatant to prepare a final concentration of 0.1 mol/L, gentle shaking to completely dissolve the PEG, and leaving overnight at 4°C. The next day, the mixture was centrifuged at 8,000 ×*g* for 30 min at 4°C, the supernatant was discarded, and the precipitate was resuspended with an appropriate amount of Tris–SM buffer to obtain a concentrated suspension of phage SauPS-28, which was then stored at 4°C. This suspension was used as the virulent species, freeze-dried, and stored at −80°C until further use.

### Host-range determination

The host range of SauPS-28 was determined using the DAP method (39). A total of 22 MRSA strains were cultured to the logarithmic growth phase (OD600 approximately 0.6), and the phage solution was serially diluted. Then, 100 µL of the bacterial solution was mixed with different titers of the phage and added to semi-solid agar at 56°C, mixed, and the surface of LB solid-agar plates was covered in an incubator at 37°C and incubated for 12 h. The formation of phage spots indicated that SauPS-28 possessed lytic activities against the strain.

### Transmission electron microscopy

A 150-mesh copper mesh with a carbon support film was used as a carrier mesh and irradiated under a UV lamp for 30 min. Then, 20 µL of a bacillus peptide was left to coat the copper mesh for 5 min and the filter paper was blotted dry. Also, 10 µL of the bacillus peptide was mixed with 10 µL of SauPS-28 and dropped onto the surface of the copper mesh and left for 20 min, after which the filter paper was blotted dry. Next, 20 µL of 2% (wt/vol) phosphotungstic acid was added to the surface for 60 s and the filter paper strips were blotted. The mesh was then placed in an airtight space and dried thoroughly. The Hitachi HT7700 TEM 80 kV was used for observation.

### Multiplicity of infection

MOI is the ratio of PFU to colony-forming units (CFU) at the onset of infection. The MOI affects the efficiency and yield of phage proliferation. Phage SauPS-28 was mixed with the MRSA bacterial solution at various MOIs (0.001, 0.01, 0.1, 1, 10, and 100) and incubated at 37°C/180 rpm for 8 hours. DAP method was used to determine the phage titers. The ideal infection multiplicity is thought to be the one that produces the offspring phage with the highest titer. The experiment was repeated thrice under the same conditions and the results are expressed as mean ± SD.

### Growth curve

The protocol of Huang et al. was employed, albeit with some minor modifications (53). The phage solution was mixed with the MRSA bacterial solution (1 × 10^8^ CFU/mL; logarithmic growth stage) at an MOI of 0.01, and incubated at 37°C for 10 min, followed by centrifugation at 4°C and 12,000 ×*g* for 1 min. The supernatant containing free phage was discarded. Next, 10 mL of the LB liquid medium was resuspended and precipitated, followed by incubation at 37°C under 180 rpm. From this, 1 mL of the sample was removed every 10 min and the whole volume was made up of an equal volume of LB medium. The samples were then centrifuged at 4°C under 12,000 ×*g* for 1 min, the supernatant was removed, and DAP method was used to determine the phage titer of the samples at each stage. Next, a one-step growth curve of phage SauPS-28 was plotted to measure the latency and burst size. The experiment was repeated thrice and the mean was taken. Data were expressed as the mean and SD.

### Acid–base stability

Different buffer pairs (such as sodium dihydrogen carbonate–disodium bicarbonate, citrate–sodium citrate, sodium hydroxide–potassium hydroxide, and sodium carbonate–bicarbonate) were adjusted to obtain buffers with pH values 1–14. The SauPS-28 suspensions were mixed with buffers of different pH values, respectively, and incubated at 37°C for 1 h. The phage titers were determined using the DAP method. The experiment was repeated thrice, and the mean and SD values were plotted.

### Thermal stability

The SauPS-28 suspensions were dispensed in centrifuge tubes and incubated at 4, 25, 37, 50, 60, and 70°C in a water bath for 0 min, 15 min, 30 min, 1 h, 2 h, 6 h, and 24 h, respectively, for the DAP determination of phage titers. The experiment was repeated thrice and plotted as the mean and SD.

### Genome extraction and restriction digest mapping

SauPS-28 genome extraction was performed in strict accordance with the procedure of the Universal Phage Genomic DNA Extraction Kit (Item No. KG005-1, Nojing, Guangdong, China). An appropriate amount of SauPS-28 genome, restriction endonuclease *EcoR I* was digested at 37°C for 4 h and then separated by 0.7% agarose gel electrophoresis.

### Phage proteome analysis

Concentrated SauPS-28 suspension (300 µL) was sonicated for 2 min at 360 W with a 10-s working interval. The protein concentration of the phage SauPS-28 was determined using the BCA method. Then, 34 µg of the phage protein was mixed with the loading buffer, boiled for 10 min, and subjected to 12% sodium dodecyl sulfate-polyacrylamide gel electrophoresis (SDS-PAGE) for separation. The BIO-RAD Molecular Imager ChemiDoc XRS + Imaging System was employed for imaging.

### Genome annotation and phylogenetic tree construction

Shanghai Heyuan Biotechnology Co., Ltd. was commissioned to complete the whole genome sequencing of phage SauPS-28 using the Illumina sequencing technology. NCBI BLASTn (http://blast.ncbi.nlm.nlm.nih.gov/) was applied for sequence identity matching and the online tool RAST (http://rast.nmpdr.org) was used for the functional annotation of ORFs. The sequences of 10 staphylococcal phage genomes with high sequence identity to SauPS-28 were downloaded, and the ClustalW comparison algorithm, maximum likelihood, based on MEGA-X software was used for sequence alignment, while a phylogenetic tree was constructed with Bootstrap repeated testing 1000 times to participate in the phylogenetic tree construction. GenBank accession numbers of the genomic sequences were as follows: B_UFSM1 (MW650841.1); B_UFSM3 (MW627293.1); B_UFSM4 (MW147366.1); StauST398-4 (KC595278.1); vB_SauS_308 (OM439664.1); B_ UFSM5 (MW192778.1); ECel-2020k (CP062426.1); ECel-2020n (CP062457.1); ECel-2020p (CP062435.1); and phiSa2wa_st59 (MG029512.1).

## References

[1] Sivori F, Cavallo I, Kovacs D, Guembe M, Sperduti I, Truglio M, Pasqua M, Prignano G, Mastrofrancesco A, Toma L, Pimpinelli F, Morrone A, Ensoli F, Di Domenico EG. 2022. Role of extracellular DNA in dalbavancin activity against methicillin-resistant Staphylococcus aureus (MRSA) biofilms in patients with skin and soft tissue infections. Microbiol Spectr 10:e0035122. doi:10.1128/spectrum.00351-22 35416701 PMC9045124

[2] Labetoulle R, Rigaill J, Lleres-Vadeboin M, Grattard F, Pozzetto B, Cazorla C, Botelho-Nevers E, Boyer B, Dupieux-Chabert C, Laurent F, Verhoeven PO, Carricajo A. 2022. Evaluation of the MRSA/SA ELITe MGB assay for the detection of Staphylococcus aureus in bone and joint infections. J Clin Microbiol 60:e0083521. doi:10.1128/JCM.00835-21 34788112 PMC8769721

[3] Cheung GYC, Bae JS, Otto M. 2021. Pathogenicity and virulence of Staphylococcus aureus. Virulence 12:547–569. doi:10.1080/21505594.2021.1878688 33522395 PMC7872022

[4] Gafter-Gvili A, Mansur N, Bivas A, Zemer-Wassercug N, Bishara J, Leibovici L, Paul M. 2011. Thrombocytopenia in Staphylococcus aureus bacteremia: risk factors and prognostic importance. Mayo Clin Proc 86:389–396. doi:10.4065/mcp.2010.0705 21531882 PMC3084641

[5] Sirobhushanam S, Parsa N, Reed TJ, Berthier CC, Sarkar MK, Hile GA, Tsoi LC, Banfield J, Dobry C, Horswill AR, Gudjonsson JE, Kahlenberg JM. 2020. Staphylococcus aureus colonization is increased on lupus skin lesions and is promoted by IFN-mediated barrier disruption. J Invest Dermatol 140:1066–1074. doi:10.1016/j.jid.2019.11.016 31877319 PMC7183889

[6] Hogan PG, Mork RL, Thompson RM, Muenks CE, Boyle MG, Sullivan ML, Morelli JJ, Williams CV, Sanchez N, Hunstad DA, Wardenburg JB, Gehlert SJ, Burnham C-A, Rzhetsky A, Fritz SA. 2020. Environmental methicillin-resistant Staphylococcus aureus contamination, persistent colonization, and subsequent skin and soft tissue infection. JAMA Pediatr 174:552–562. doi:10.1001/jamapediatrics.2020.0132 32227144 PMC7105954

[7] Argudín MÁ, Mendoza MC, Rodicio MR. 2010. Food poisoning and Staphylococcus aureus enterotoxins. Toxins (Basel) 2:1751–1773. doi:10.3390/toxins2071751 22069659 PMC3153270

[8] Hennekinne J-A, De Buyser M-L, Dragacci S. 2012. Staphylococcus aureus and its food poisoning toxins: characterization and outbreak investigation. FEMS Microbiol Rev 36:815–836. doi:10.1111/j.1574-6976.2011.00311.x 22091892

[9] Jeyanthi V, Velusamy P, Kumar GV, Kiruba K. 2021. Effect of naturally isolated hydroquinone in disturbing the cell membrane integrity of Pseudomonas aeruginosa MTCC 741 and Staphylococcus aureus MTCC 740. Heliyon 7:e07021. doi:10.1016/j.heliyon.2021.e07021 34036196 PMC8134992

[10] Chi C-Y, Wang S-M, Lin H-C, Liu C-C. 2006. A clinical and microbiological comparison of Staphylococcus aureus toxic shock and scalded skin syndromes in children. Clin Infect Dis 42:181–185. doi:10.1086/498901 16355327

[11] Courjon J, Hubiche T, Phan A, Tristan A, Bès M, Vandenesch F, Etienne J, Del Giudice P, Gillet Y. 2013. Skin findings of Staphylococcus aureus toxin-mediated infection in relation to toxin encoding genes. Pediatr Infect Dis J 32:727–730. doi:10.1097/INF.0b013e31828e89f5 23446443

[12] Hatfull GF, Hendrix RW. 2011. Bacteriophages and their genomes. Curr Opin Virol 1:298–303. doi:10.1016/j.coviro.2011.06.009 22034588 PMC3199584

[13] Ge H, Zhang K, Gu D, Chen X, Wang X, Li G, Zhu H, Chang Y, Zhao G, Pan Z, Jiao X, Hu M. 2021. The rfbN gene of Salmonella typhimurium mediates phage adsorption by modulating biosynthesis of lipopolysaccharide. Microbiol Res 250:126803. doi:10.1016/j.micres.2021.126803 34146940

[14] Ge H, Fu S, Guo H, Hu M, Xu Z, Zhou X, Chen X, Jiao X. 2022. Application and challenge of bacteriophage in the food protection. Int J Food Microbiol 380:109872. doi:10.1016/j.ijfoodmicro.2022.109872 35981493

[15] Jamal M, Bukhari S, Andleeb S, Ali M, Raza S, Nawaz MA, Hussain T, Rahman SU, Shah SSA. 2019. Bacteriophages: an overview of the control strategies against multiple bacterial infections in different fields. J Basic Microbiol 59:123–133. doi:10.1002/jobm.201800412 30485461

[16] Zhang G, Zhao Y, Paramasivan S, Richter K, Morales S, Wormald P-J, Vreugde S. 2018. Bacteriophage effectively kills multidrug resistant Staphylococcus aureus clinical isolates from chronic rhinosinusitis patients. Int Forum Allergy Rhinol 8:406–414. doi:10.1002/alr.22046 29240296

[17] Chanishvili N. 2012. Phage therapy--history from Twort and d'Herelle through soviet experience to current approaches. Adv Virus Res 83:3–40. doi:10.1016/B978-0-12-394438-2.00001-3 22748807

[18] Anyaegbunam NJ, Anekpo CC, Anyaegbunam ZKG, Doowuese Y, Chinaka CB, Odo OJ, Sharndama HC, Okeke OP, Mba IE. 2022. The resurgence of phage-based therapy in the era of increasing antibiotic resistance: from research progress to challenges and prospects. Microbiol Res 264:127155. doi:10.1016/j.micres.2022.127155 35969943

[19] Touchon M, Bernheim A, Rocha EP. 2016. Genetic and life-history traits associated with the distribution of prophages in bacteria. ISME J 10:2744–2754. doi:10.1038/ismej.2016.47 27015004 PMC5113838

[20] Dedrick RM, Jacobs-Sera D, Bustamante CAG, Garlena RA, Mavrich TN, Pope WH, Reyes JCC, Russell DA, Adair T, Alvey R, et al.. 2017. Prophage-mediated defence against viral attack and viral counter-defence. Nat Microbiol 2:16251. doi:10.1038/nmicrobiol.2016.251 28067906 PMC5508108

[21] Harrison E, Brockhurst MA. 2017. Ecological and evolutionary benefits of temperate phage: what does or doesn't kill you makes you stronger. Bioessays 39. doi:10.1002/bies.201700112 28983932

[22] Davies EV, Winstanley C, Fothergill JL, James CE. 2016. The role of temperate bacteriophages in bacterial infection. FEMS Microbiol Lett 363:fnw015. doi:10.1093/femsle/fnw015 26825679

[23] Shaikh N, Tarr PI. 2003. Escherichia coli O157:H7 shiga toxin-encoding bacteriophages: integrations, excisions, truncations, and evolutionary implications. J Bacteriol 185:3596–3605. doi:10.1128/JB.185.12.3596-3605.2003 12775697 PMC156235

[24] Waldor MK, Mekalanos JJ. 1996. Lysogenic conversion by a filamentous phage encoding cholera toxin. Science 272:1910–1914. doi:10.1126/science.272.5270.1910 8658163

[25] Haaber J, Leisner JJ, Cohn MT, Catalan-Moreno A, Nielsen JB, Westh H, Penadés JR, Ingmer H. 2016. Bacterial viruses enable their host to acquire antibiotic resistance genes from neighbouring cells. Nat Commun 7:13333. doi:10.1038/ncomms13333 27819286 PMC5103068

[26] Chen J, Novick RP. 2009. Phage-mediated intergeneric transfer of toxin genes. Science 323:139–141. doi:10.1126/science.1164783 19119236

[27] Dearborn AD, Dokland T. 2012. Mobilization of pathogenicity islands by Staphylococcus aureus strain newman bacteriophages. Bacteriophage 2:70–78. doi:10.4161/bact.20632 23050217 PMC3442828

[28] Hargreaves KR, Clokie MRJ. 2014. Clostridium difficile phages: still difficult? Front Microbiol 5:184. doi:10.3389/fmicb.2014.00184 24808893 PMC4009436

[29] Zhang H, Fouts DE, DePew J, Stevens RH. 2013. Genetic modifications to temperate Enterococcus faecalis phage Ef11 that abolish the establishment of lysogeny and sensitivity to repressor, and increase host range and productivity of lytic infection. Microbiology (Reading) 159:1023–1035. doi:10.1099/mic.0.067116-0 23579685 PMC3709695

[30] Kilcher S, Studer P, Muessner C, Klumpp J, Loessner MJ. 2018. Cross-genus rebooting of custom-made, synthetic bacteriophage genomes in L-form bacteria. Proc Natl Acad Sci USA 115:567–572. doi:10.1073/pnas.1714658115 29298913 PMC5776983

[31] Park JY, Moon BY, Park JW, Thornton JA, Park YH, Seo KS. 2017. Genetic engineering of a temperate phage-based delivery system for CRISPR/Cas9 antimicrobials against Staphylococcus aureus. Sci Rep 7:44929. doi:10.1038/srep44929 28322317 PMC5359561

[32] Yosef I, Manor M, Kiro R, Qimron U. 2015. Temperate and lytic bacteriophages programmed to sensitize and kill antibiotic-resistant bacteria. Proc Natl Acad Sci USA 112:7267–7272. doi:10.1073/pnas.1500107112 26060300 PMC4466736

[33] Whittard E, Redfern J, Xia G, Millard A, Ragupathy R, Malic S, Enright MC. 2021. Phenotypic and genotypic characterization of novel polyvalent bacteriophages with potent in vitro activity against an international collection of genetically diverse Staphylococcus aureus. Front Cell Infect Microbiol 11:698909. doi:10.3389/fcimb.2021.698909 34295840 PMC8290860

[34] Doub JB, Ng VY, Lee M, Chi A, Lee A, Würstle S, Chan B. 2022. Salphage: salvage bacteriophage therapy for recalcitrant MRSA prosthetic joint infection. Antibiotics (Basel) 11:616. doi:10.3390/antibiotics11050616 35625260 PMC9137795

[35] Ferry T, Kolenda C, Batailler C, Gaillard R, Gustave C-A, Lustig S, Fevre C, Petitjean C, Leboucher G, Laurent F, Lyon BJI Study group. 2021. Case report: arthroscopic "Debridement antibiotics and implant retention" with local injection of personalized phage therapy to salvage a relapsing Pseudomonas aeruginosa prosthetic knee infection. Front Med (Lausanne) 8:569159. doi:10.3389/fmed.2021.569159 34026768 PMC8132876

[36] Kosznik-Kwaśnicka K, Stasiłojć M, Grabowski Ł, Zdrojewska K, Węgrzyn G, Węgrzyn A. 2022. Efficacy and safety of phage therapy against Salmonella enterica serovars typhimurium and enteritidis estimated by using a battery of in vitro tests and the Galleria mellonella animal model. Microbiol Res 261:127052. doi:10.1016/j.micres.2022.127052 35533436

[37] Xu Z, Shao S, Ding Z, Zhang Y, Wang Q, Liu X, Liu Q. 2022. Therapeutic efficacies of two newly isolated Edwardsiella phages against Edwardsiella piscicida infection. Microbiol Res 263:127043. doi:10.1016/j.micres.2022.127043 35834890

[38] Schoeffel J, Wang EW, Gill D, Frackler J, Horne B, Manson T, Doub JB. 2022. Successful use of salvage bacteriophage therapy for a recalcitrant MRSA knee and hip prosthetic joint infection. Pharmaceuticals (Basel) 15:177. doi:10.3390/ph15020177 35215290 PMC8877365

[39] Teng F, Xiong X, Zhang S, Li G, Wang R, Zhang L, Wang X, Zhou H, Li J, Li Y, Jiang Y, Cui W, Tang L, Wang L, Qiao X. 2022. Efficacy assessment of phage therapy in treating Staphylococcus aureus-Induced mastitis in mice. Viruses 14:620. doi:10.3390/v14030620 35337027 PMC8954217

[40] Chung I-Y, Sim N, Cho Y-H. 2012. Antibacterial efficacy of temperate phage-mediated inhibition of bacterial group motilities. Antimicrob Agents Chemother 56:5612–5617. doi:10.1128/AAC.00504-12 22908158 PMC3486593

[41] Breyne K, Honaker RW, Hobbs Z, Richter M, Żaczek M, Spangler T, Steenbrugge J, Lu R, Kinkhabwala A, Marchon B, Meyer E, Mokres L. 2017. Efficacy and safety of a bovine-associated Staphylococcus aureus phage cocktail in a murine model of mastitis. Front Microbiol 8:2348. doi:10.3389/fmicb.2017.02348 29234314 PMC5712351

[42] Kebriaei R, Lev KL, Shah RM, Stamper KC, Holger DJ, Morrisette T, Kunz Coyne AJ, Lehman SM, Rybak MJ. 2022. Eradication of biofilm-mediated methicillin-resistant Staphylococcus aureus infections in vitro: bacteriophage-antibiotic combination. Microbiol Spectr 10:e0041122. doi:10.1128/spectrum.00411-22 35348366 PMC9045164

[43] Rodriguez JM, Woodworth BA, Horne B, Fackler J, Brownstein MJ. 2022. Case report: successful use of phage therapy in refractory MRSA chronic rhinosinusitis. Int J Infect Dis 121:14–16. doi:10.1016/j.ijid.2022.04.049 35472526

[44] Adriaenssens EM, Edwards R, Nash JHE, Mahadevan P, Seto D, Ackermann H-W, Lavigne R, Kropinski AM. 2015. Integration of genomic and proteomic analyses in the classification of the siphoviridae family. Virology 477:144–154. doi:10.1016/j.virol.2014.10.016 25466308

[45] Ferry T, Kolenda C, Laurent F, Leboucher G, Merabischvilli M, Djebara S, Gustave C-A, Perpoint T, Barrey C, Pirnay J-P, Resch G. 2022. Personalized bacteriophage therapy to treat pandrug-resistant spinal Pseudomonas aeruginosa infection. Nat Commun 13:4239. doi:10.1038/s41467-022-31837-9 35869081 PMC9306240

[46] Forti F, Roach DR, Cafora M, Pasini ME, Horner DS, Fiscarelli EV, Rossitto M, Cariani L, Briani F, Debarbieux L, Ghisotti D. 2018. Design of a broad-range bacteriophage cocktail that reduces Pseudomonas aeruginosa biofilms and treats acute infections in two animal models. Antimicrob Agents Chemother 62:e02573-17. doi:10.1128/AAC.02573-17 29555626 PMC5971607

[47] Cui Z, Guo X, Dong K, Zhang Y, Li Q, Zhu Y, Zeng L, Tang R, Li L. 2017. Safety assessment of Staphylococcus phages of the family myoviridae based on complete genome sequences. Sci Rep 7:41259. doi:10.1038/srep41259 28117392 PMC5259776

[48] Touchon M, Moura de Sousa JA, Rocha EP. 2017. Embracing the enemy: the diversification of microbial gene repertoires by phage-mediated horizontal gene transfer. Curr Opin Microbiol 38:66–73. doi:10.1016/j.mib.2017.04.010 28527384

[49] Monteiro R, Pires DP, Costa AR, Azeredo J. 2019. Phage therapy: going temperate? Trends Microbiol 27:368–378. doi:10.1016/j.tim.2018.10.008 30466900

[50] Lenneman BR, Fernbach J, Loessner MJ, Lu TK, Kilcher S. 2021. Enhancing phage therapy through synthetic biology and genome engineering. Curr Opin Biotechnol 68:151–159. doi:10.1016/j.copbio.2020.11.003 33310655 PMC11996084

[51] Shin H, Lee J-H, Yoon H, Kang D-H, Ryu S. 2014. Genomic investigation of lysogen formation and host lysis systems of the salmonella temperate bacteriophage SPN9CC. Appl Environ Microbiol 80:374–384. doi:10.1128/AEM.02279-13 24185850 PMC3911004

[52] Dedrick RM, Guerrero-Bustamante CA, Garlena RA, Russell DA, Ford K, Harris K, Gilmour KC, Soothill J, Jacobs-Sera D, Schooley RT, Hatfull GF, Spencer H. 2019. Engineered bacteriophages for treatment of a patient with a disseminated drug-resistant Mycobacterium abscessus. Nat Med 25:730–733. doi:10.1038/s41591-019-0437-z 31068712 PMC6557439

[53] Huang C, Shi J, Ma W, Li Z, Wang J, Li J, Wang X. 2018. Isolation, characterization, and application of a novel specific Salmonella bacteriophage in different food matrices. Food Res Int 111:631–641. doi:10.1016/j.foodres.2018.05.071 30007727

